# Genetic rearrangements, hotspot mutations, and microRNA expression in the progression of metastatic adenoid cystic carcinoma of the salivary gland

**DOI:** 10.18632/oncotarget.24800

**Published:** 2018-04-13

**Authors:** Simon Andreasen, Tina Klitmøller Agander, Kristine Bjørndal, Daiva Erentaite, Steffen Heegaard, Stine R. Larsen, Linea Cecilie Melchior, Qihua Tan, Benedicte Parm Ulhøi, Irene Wessel, Preben Homøe

**Affiliations:** ^1^ Department of Otorhinolaryngology and Maxillofacial Surgery, Zealand University Hospital, Køge, Denmark; ^2^ Department of Otorhinolaryngology Head and Neck Surgery and Audiology, Rigshospitalet, Copenhagen, Denmark; ^3^ Department of Pathology, Rigshospitalet, Copenhagen, Denmark; ^4^ Department of Otorhinolaryngology, Head and Neck Surgery, Odense University Hospital, Odense, Denmark; ^5^ Department of Pathology, Aalborg University Hospital, Aalborg, Denmark; ^6^ Department of Ophthalmology, Rigshospitalet-Glostrup, Copenhagen, Denmark; ^7^ Department of Pathology, Odense University Hospital, Odense, Denmark; ^8^ Unit of Human Genetics, Department of Clinical Research, University of Southern Denmark, Odense, Denmark; ^9^ Department of Pathology, Aarhus University Hospital, Aarhus, Denmark

**Keywords:** adenoid cystic carcinoma, salivary gland, metastases, MYB, microRNA

## Abstract

Adenoid cystic carcinoma (ACC) is among the most common salivary gland malignancies, and is notorious for its unpredictable clinical course with frequent local recurrences and metastatic spread. However, the molecular mechanisms for metastatic spread are poorly understood. This malignancy is known to frequently harbor gene fusions involving *MYB*, *MYBL1*, and *NFIB*, and to have a low mutational burden. Most studies have focused on primary tumors to understand the biology of ACC, but this has not revealed a genetic cause for metastatic dissemination in the majority of cases. Hence, other molecular mechanisms are likely to be involved. Here, we characterize the genetic and microRNA expressional landscape of primary ACC and corresponding metastatic lesions from 11 patients. FISH demonstrated preservation of *MYB* aberrations between primary tumors and metastases, and targeted next-generation sequencing identified mutations exclusive for the metastatic lesions in 3/11 cases (27.3%). Global microRNA profiling identified several differentially expressed miRNAs between primary ACC and metastases as compared to normal salivary gland tissue. Interestingly, individual tumor pairs differed in miRNA profile, but there was no general difference between primary ACCs and metastases. Collectively, we show that *MYB* and *NFIB* aberrations are consistently preserved in ACC metastatic lesions, and that additional mutations included in the 50-gene hotspot panel used are infrequently acquired by the metastatic lesions. In contrast, tumor pairs differ in microRNA expression and our data suggest that they are heterogeneous according to their microRNA profile. This adds an additional layer to the complex process of ACC metastatic spread.

## INTRODUCTION

Metastatic dissemination is a fundamental process in the progression of malignant disease, and is the cause of the majority of cancer-related deaths [1]. Despite this the mechanisms underlying the acquired ability of cells to metastasize are poorly understood [2]. As a consequence, the ability to accurately predict, prevent, and treat metastatic disease remain a major challenge in prognostication and oncological treatment. However, while most studies explore primary tumors in the search for molecular answers to these challenges, identification of discordances between primary tumors and metastases have the potential to reveal key drivers of metastatic progression.

Adenoid cystic carcinoma (ACC) is among the most frequent and enigmatic malignancies of the salivary gland [3]. It is characterized by an unpredictable clinical course with frequent local recurrences, late onset of metastases mainly to the lungs and liver and, consequently, an often fatal outcome [4, 5]. The genetics of primary ACC have been extensively explored, which has resulted in the identification of recurrent gene fusions involving the *MYB*, *MYBL1*, and *NFIB* genes and frequent mutations in NOTCH pathway genes [6–8]. So far, these characteristic genetic profiles have provided a valuable insight into the biology of ACC but have not contributed to the understanding of metastatic dissemination in the majority of patients [9, 10]. Recently, spatial genetic differences within and between paired primary ACCs and metastases have been reported, but only few and diverse mutations were found in coding regions [11]. While this study demonstrates the relatively quiet genome of primary ACC is preserved in metastases, it provides explanations to metastatic spread in a subset of cases but at the same time raises the question as to whether additional molecular mechanisms are involved in metastatic spread of ACC.

MicroRNAs (miRNAs) are short non-coding RNAs ∼22 nucleotides in length, functioning as potent post-transcriptional regulators of gene expression [12]. MiRNAs have been shown to be implicated in numerous cancers and to be of prognostic value, including in salivary gland ACC [13, 14]. Several *in vitro* studies have characterized the effects of miRNAs on primary and metastatic ACC cell lines, but the findings have recently been discredited due to contamination of all of these cultures with various non-ACC cell lines [15]. Hence, reliable data from human ACC samples is highly warranted.

To further explore the molecular background for ACC dissemination, we performed fluorescence *in situ* hybridization (FISH), targeted next-generation sequencing (NGS), and global miRNA expression profiling in paired samples of primary and metastatic ACC.

## RESULTS

### Clinicopathologic characteristics

**Figure 1:**
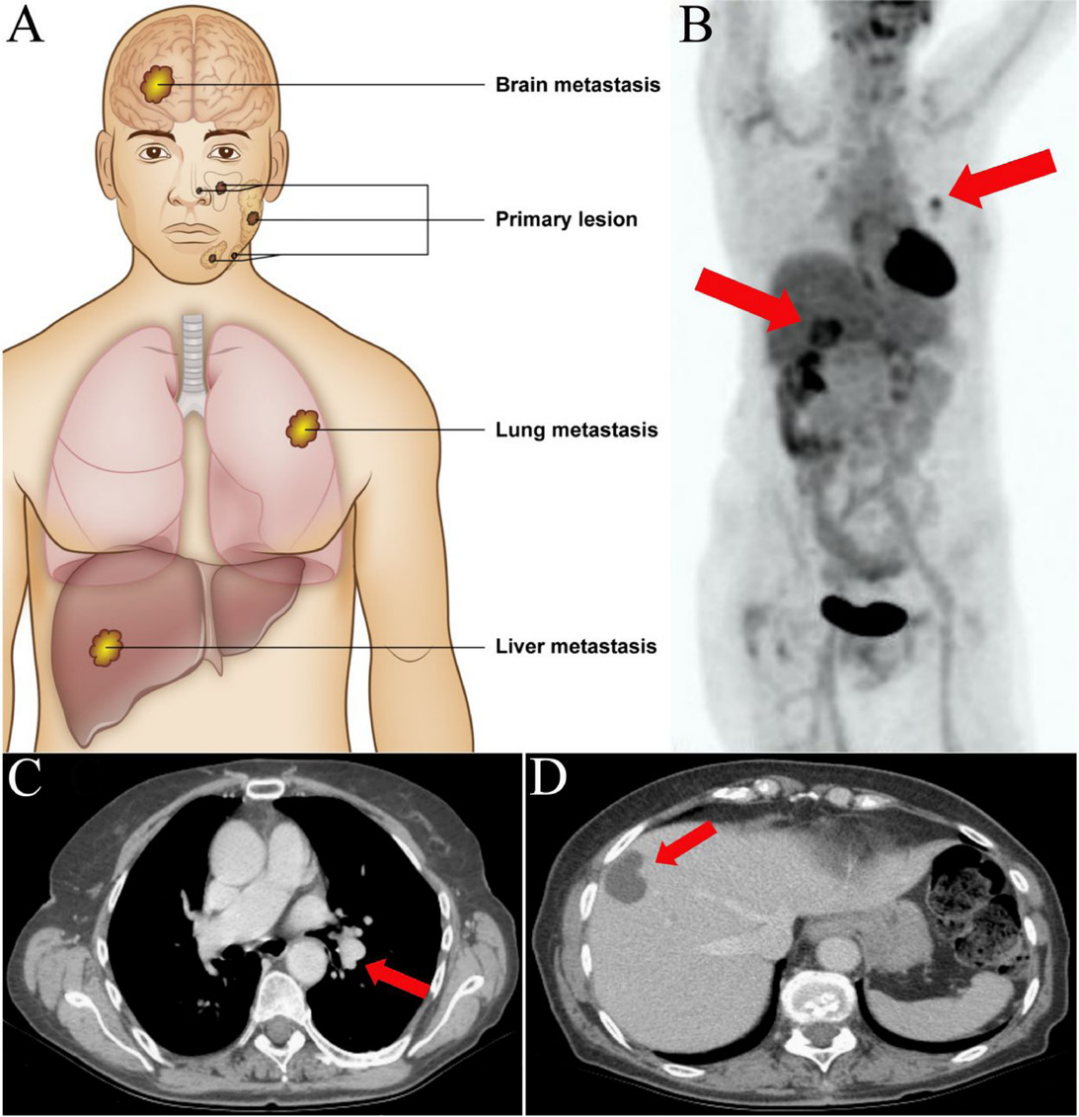
Anatomical localization of primary salivary gland adenoid cystic carcinomas and distant metastases (**A**) Primary adenoid cystic carcinomas were located to the major and minor salivary glands of the head and neck. Distant metastases were located to the brain (*n* = 1), lungs (*n* = 8), and liver (*n* = 3). (**B**) Positron emission tomography – computerized tomography (PET-CT) scan of case 6 with metachronous metastases to the hilar region of the left lung and liver (red arrows). (**C**) CT-scan of the same patient showing the pulmonary and (**D**) hepatic metastases.

Patients included six males and five females with primary ACC of various head and neck sites (Table 1, Figure 1A). Radical surgical removal of the primary lesion was performed in 4/11 primary tumors. All metastases were metachronous and were from brain (case 1), lung (cases 2–9), and liver (case 5, 10, and 11), with latency from surgery of the primary lesion to metastatic spread ranging from 7–119 months (median: 60.5) (Figure 1B–1D). Radical surgical removal of the metastatic lesion was performed in 9/11 patients. Median follow-up was 46 months (range: 2–97). At the end of follow-up, five patients had died of disseminated disease, three were alive with disease, and three had no evidence of disease. Clinicopathologic characteristics of the 25 patients without recurrence and 11 additional patients with metastatic spread are listed in Supplementary Table 1.

**Table 1:** Clinicopathologic characteristics at diagnosis of primary and metastatic adenoid cystic carcinoma

Case	Age/Sex	Site of primary	RT	Histology	TNM at diagnosis	Stage at diagnosis	Radical	Time to metastasis, months	Site of metastasis	Histology	Radical	Outcome, months^^^
1	47/M	Submandibular	+	S	T1N1M0	I	No	119	Brain	S	No	DOD, 2
2	53/M	Submandibular	+	TC	T1N0M0	I	No	52	Lung	TC	Yes	NED, 19
3	53/M	Submandibular	+	TC	T2N3M0	IVB	No	11	Lung	TC	No	DOD, 3
4	57/M	Base of tongue	+	TC	T3N0M0	III	No	7	Lung	TC	Yes	AWD, 36
5	36/F	Hard palate	+	TC	T1N0M0	I	No	69	Lung	TC	Yes	DOD, 90
6	58/F	Sinus	+	TC	T2N0M0	II	No	107 114	Liver Lung	S/TC^*^ TC	Yes Yes	AWD, 26
7	49/M	Base of tongue	+	TC	T3N0M0	III	No	45	Lung	TC	Yes	DOD, 46
8	67/M	Parotid	+	S	T2N0M0	II	Yes	9	Lung	S	Yes	DOD, 73
9	63/F	Parotid	+	TC	T4aN0M0	IVA	Yes	68	Lung	TC	Yes	NED, 75
10	59/F	Parotid	+	TC	T2N0M0	II	Yes	102	Liver	TC	Yes	AWD, 97
11	62/F	Sublingual	+	TC	T2N1M0	III	Yes	53	Liver	TC	Yes	NED, 52

^^^After diagnosis of metastatic disease. ^*^multiple metastases of which some were solid and others tubulocribriform. RT = radiotherapy; S = solid; TC = tubulocribriform; DOD = Dead of disease; NED = No evidence of disease; AWD = Alive with disease.

### Histopathology

**Figure 2:**
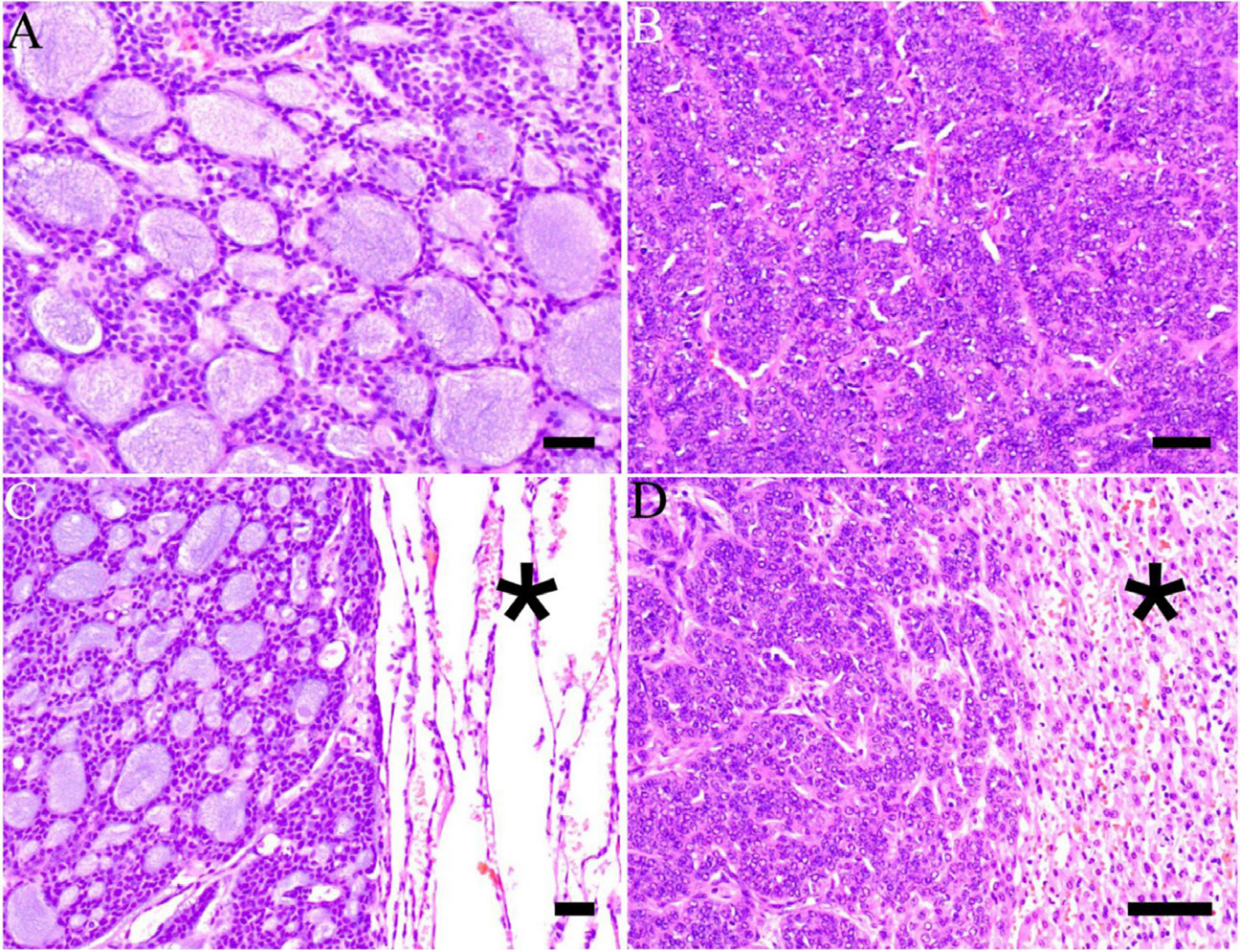
Histological features of primary and metastatic salivary gland adenoid cystic carcinoma (**A**) Adenoid cystic carcinoma of the sinonasal minor salivary glands with tubular and cribriform areas and accumulation of basophilic basal membrane material in pseudoluminae (case 6) (hematoxylin & eosin, H&E). (**B**) Adenoid cystic carcinoma of the submandibular gland from case 1 with solid aggregates of basaloid tumor cells. Nuclei are large and mitoses are frequent (H&E). (**C**) Pulmonary metastasis of a well-differentiated tubulocribriform adenoid cystic carcinoma from the primary tumor in (A). Normal lung parenchyma is seen to the right (asterisk) (H&E). (**D**) Hepatic metastasis showing a solid adenoid cystic carcinoma. This specimen is from the same patient as in (A and C). Normal liver parenchyma is seen to the right (asterisk) (H&E). Scale bar = 100 μm.

**Figure 3:**
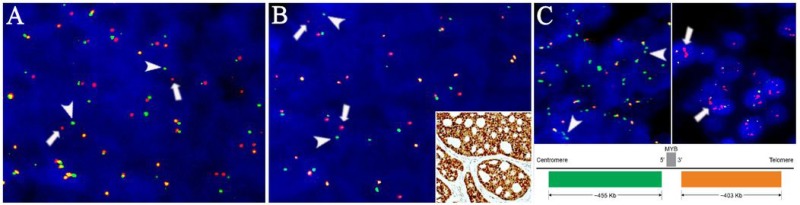
Rearrangements and copy number status of *MYB* and *NFIB* in primary and metastatic adenoid cystic carcinoma Fluorescence *in situ* hybridization with break-apart probes showing split signals of the green centromeric (arrowheads) and red telomeric (arrows) for (**A**) *MYB* and (**B**) *NFIB* in a pulmonary metastasis. (B, insert) Representative area of the primary tumor in case 5 showing diffuse positivity for MYB protein despite being without detectable *MYB* abnormalities. (**C**, upper left) *MYB* break-apart probe showing isolated amplification of the green centromeric probe (arrowheads, case 3) and (C, upper right) red telomeric probe (arrows, case 6). (C, bottom) Schematic presentation of the chromosomal location of the break-apart probe. Multiple green and red signals are consistent with isolated amplification of the 5′ and 3′ parts of the *MYB* gene, respectively. Image is not drawn to scale.

Histologically, the majority of primary ACCs (9/11) displayed tubulocribriform growth patterns with dual populations of luminal and abluminal cells, whereas two cases were of solid type (Figure 2A, 2B). The growth pattern between primary tumors and metastases was preserved in all but one case in which the primary tumor was tubulocribriform and some of the multiple metastases were of solid type (case 6) (Table 1) (Figure 2C, 2D). Immunohistochemically, all primary ACCs and metastases were positive for CK7, CD117, and MYB (Figure 3B, insert) and consistently negative for CK20 and neuroendocrine markers (CD56, chromogranin A). Dual TTF-1 and napsin A positivity was focal in pulmonary metastases of two cases. Ki-67 indices in primary tumors and metastases ranged from 5–60% (median: 10). All immunohistochemical results are summarized in Supplementary Table 2.

### FISH

In all cases, the pattern of *MYB* and *NFIB* aberrations was identical between primary ACC and metastasis (Table 2). In 4/10 patients, concurrent rearrangements of *MYB* and *NFIB* were identified (Figure 3A, 3B). In the majority of tumor cells, the MYB break-apart probe showed a green/red signal ratio of 3 in case 3 and a red/green signal ratio of 3 in case 6, consistent with amplification of the 5′ and 3′ parts of MYB, respectively (Figure 3C). Four cases with MYB protein expression did not harbor identifiable alterations in *MYB* (Figure 3B, insert and Table 2). *MYBL1* was intact in all cases (not shown).

**Table 2:** Rearrangement of *MYB* and *NFIB* in primary adenoid cystic carcinoma and metastases

Case	Primary adenoid cystic carcinoma	Metastatic adenoid cystic carcinoma
1	*MYB-NFIB*	*MYB-NFIB*
2	Normal	Normal
3	*MYB* amplification^1^	*MYB* amplification^1^
4	*MYB-NFIB*	*MYB-NFIB*
5	Normal	Normal
6	*MYB* amplification^2^	Liver *MYB* amplification^2^
		Lung *MYB* amplification^2^
7	*MYB-NFIB*	*MYB-NFIB*
8	*MYB-NFIB*	*MYB-NFIB*
9	Normal	Lung^3^ Normal
		Lung^3^ Normal
10	Not available	Not available
11	Normal	Normal

All patients had intact *MYBL1* genes.^1^Multiple green signals per nucleus, consistent with 5′ amplification. ^2^Multiple red signals per nucleus, consistent with 3′ amplification. ^3^Separate tubulocribriform metastases from right and left lung, respectively.

### Targeted next-generation sequencing of primary and metastatic tumor tissue

Point mutations in primary and metastatic ACC was assessed using a targeted NGS panel covering approximately 2,800 mutational hotspots in 50 cancer-related oncogenes and tumor-supressor genes. Truncal mutations present in both primary tumor and metastasis were identified in case 1 (*NRAS*, *NOTCH1*), case 4 (*BRAF*, *TP53*), and case 11 (*APC*). In wild-type primary tumors, metastases showed single mutations in case 3 (*NRAS*), case 6 (*PIK3CA* in lung metastasis and *BRAF* in liver metastasis), and case 8 (*HRAS*) (Table 3). Loss of mutations from primary tumor to metastases were identified in case 5 (*PDGFRA*) and case 9 (*FGFR2*). No mutations were identified in 6/11 (54.5%) of primary ACCs and three of these had no mutations in the metastatic lesions. In order to account for patient-specific single nucleotide polymorphisms (SNPs), matched normal salivary gland tissue was sequenced from the seven cases with this material available. This did not exclude any of the mutations identified in neither primary ACCs or distant metastases.

**Table 3:** Hotspot mutations in paired primary and metastatic salivary gland adenoid cystic carcinoma

Case	Normal salivary gland	Primary adenoid cystic carcinoma				Metastasis			
	Mutation	Location	Gene	Nucleotide change (frequency)	Amino acid change	Location	Gene	Nucleotide change (frequency)	Amino acid change
1	No mutation	Submandibular	*NRAS* *NOTCH1* *TP53*	c.35G>A (7.4) c.7408delT (48.4) 818G>A (11.3)	p.G12D^#^ p.S2470fs* 7p.R273H^#^	Brain	*NRAS* *NOTCH1*	c.35G>A (47.1) c.7408delT (41.5)	p.G12D^#^ p.S2470fs*7
2	No mutation	Submandibular	WT			Lung	WT		
3	No mutation	Submandibular	WT			Lung	*NRAS*	c.35G>A (35.8)	p.G12D^#^
4	-	Base of tongue	*BRAF* *TP53*	c.1742A>T (44.6) c.844C>T (44.1)	p.N581I^#^ p.R282W^#^	Lung	*BRAF* *TP53*	c.1742A>T (33) c.844C>T (35.5)	p.N581I^#^ p.R282W^#^
5	-	Hard palate	*PDGFRA*	c.2546A>G (9.3)	p.Y849C	Lung	WT		
6	-	Sinus	WT			Lung	*PIK3CA*	c.1624G>A (31.9)	p.E542K^#^
						Liver	*BRAF*	c.1780G>A (47.2)	p.D594N^#^
7	-	Base of tongue	WT			Lung	WT		
8	No mutation	Parotid	WT			Lung	*HRAS*	c.37G>C (44.8)	p.G13R^#^
9	No mutation	Parotid	*FGFR2*	c.1150G>A (11.5)	p.G384R	Lung^R1^	WT		
						Lung^L1^	WT		
10	No mutation	Sublingual	WT			Liver	WT		
11	No mutation	Parotid	*APC*	c.3313C>T (51.2)	p.R1105W	Liver	*APC*	c.3313C>T (53.2)	p.R1105W

^1^Separate tubulocribriform metastases from right and left lung, respectively. ^#^Recurrent oncogenic hotspot mutation.

### MicroRNA expression profiling of primary and metastatic tumor tissue

**Figure 4:**
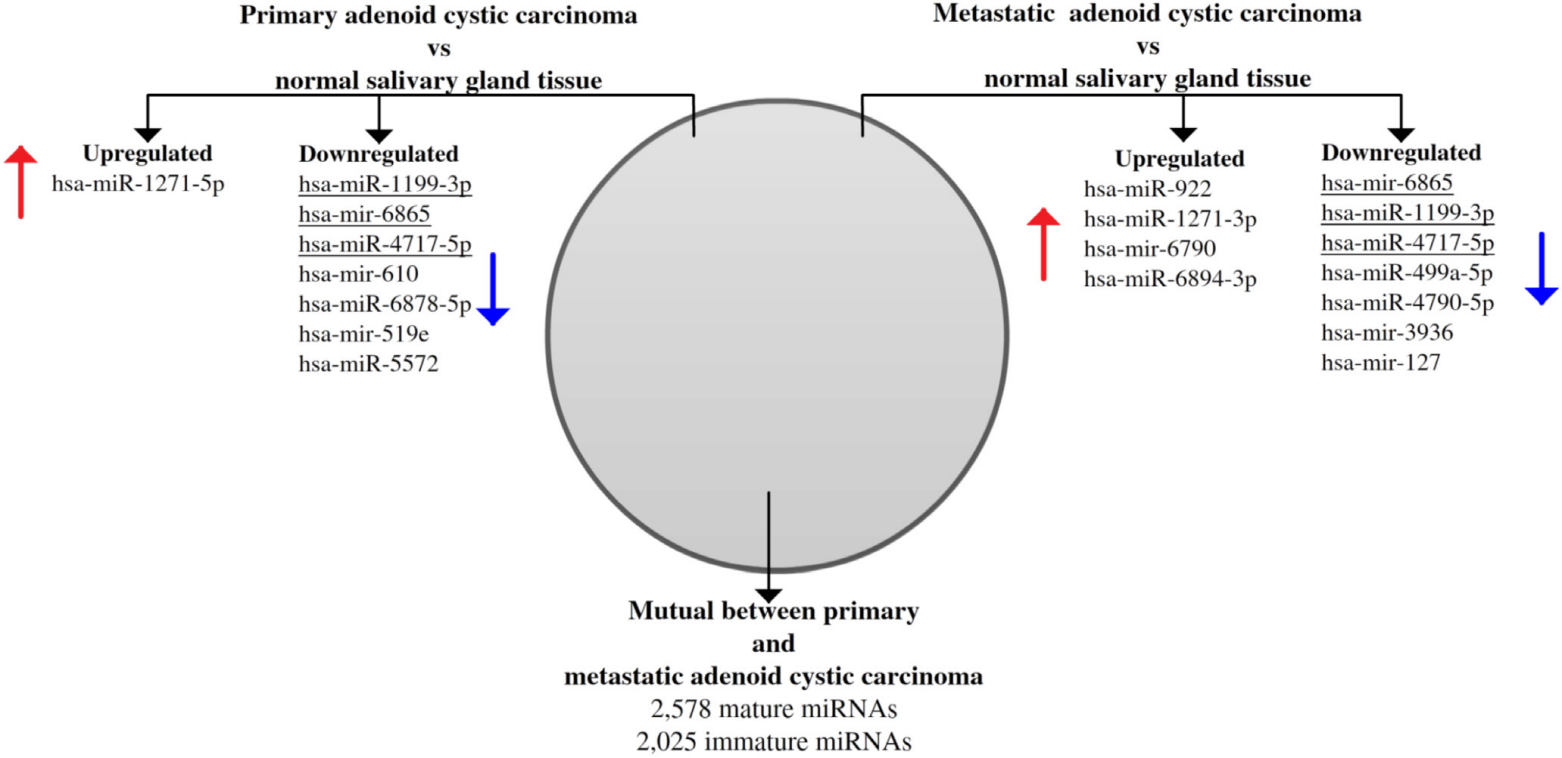
Venn diagram showing differentially expressed microRNAs between normal salivary gland tissue as compared to primary and metastatic adenoid cystic carcinoma Eight miRNAs were differentially expressed between normal salivary gland tissue and primary adenoid cystic carcinoma, and 11 miRNAs were differentially expressed between normal salivary gland tissue and distant metastases. Three miRNAs were downregulated in both tumor tissues (underlined). There was no difference between primary tumor and metastases, and therefore all miRNAs investigated were mutual.

**Figure 5:**
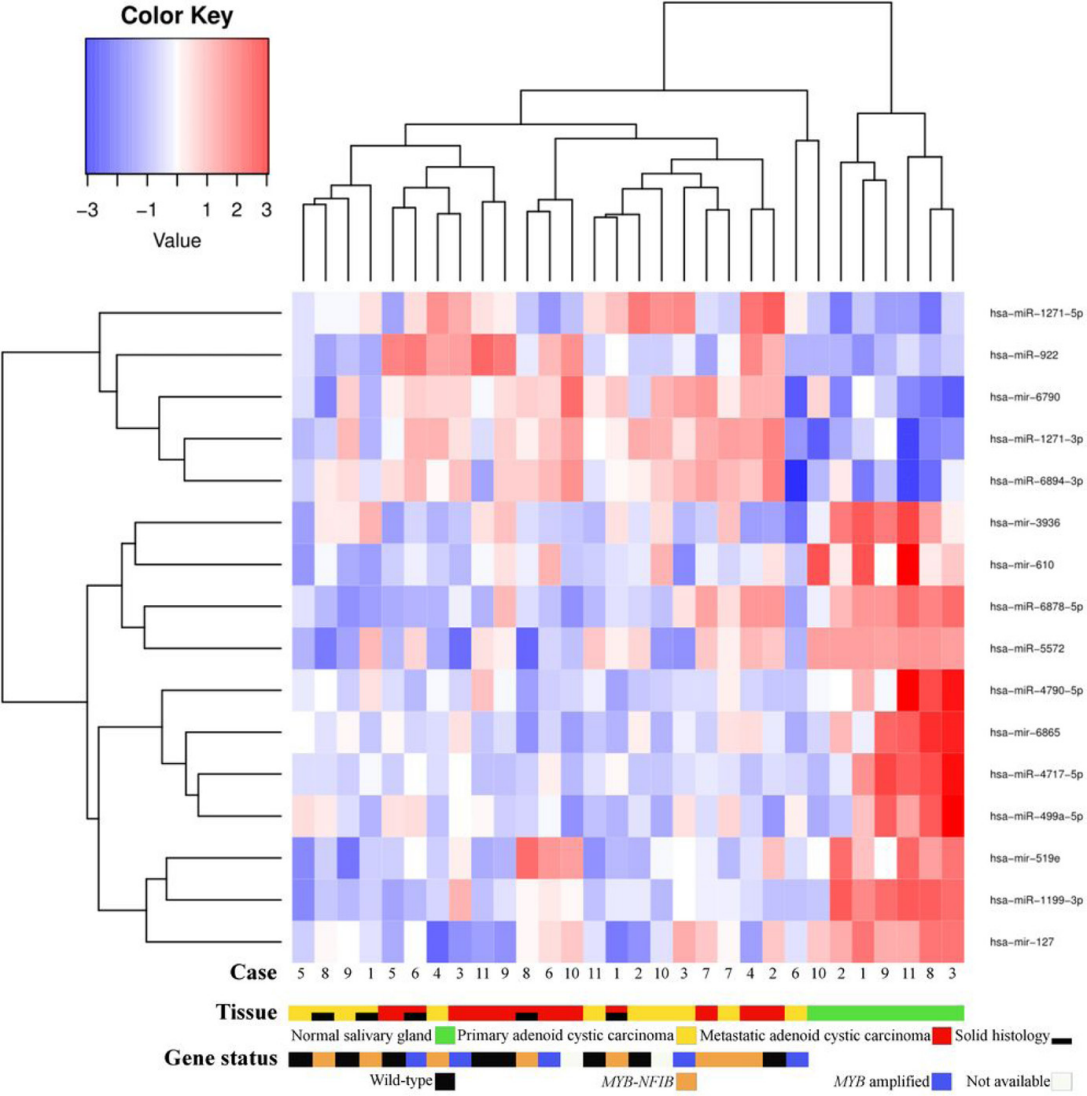
Heatmap of microRNA expression in primary adenoid cystic carcinoma, metastatic adenoid cystic carcinoma, and normal salivary gland tissue Normal salivary gland tissue is clustered to the right. No difference in microRNA expression was found between primary tumors and metastases. In the dendrogram, primary tumors and corresponding metastases were only highly similar in case 7. There was no clustering of cases according to gene fusion status.

**Figure 6:**
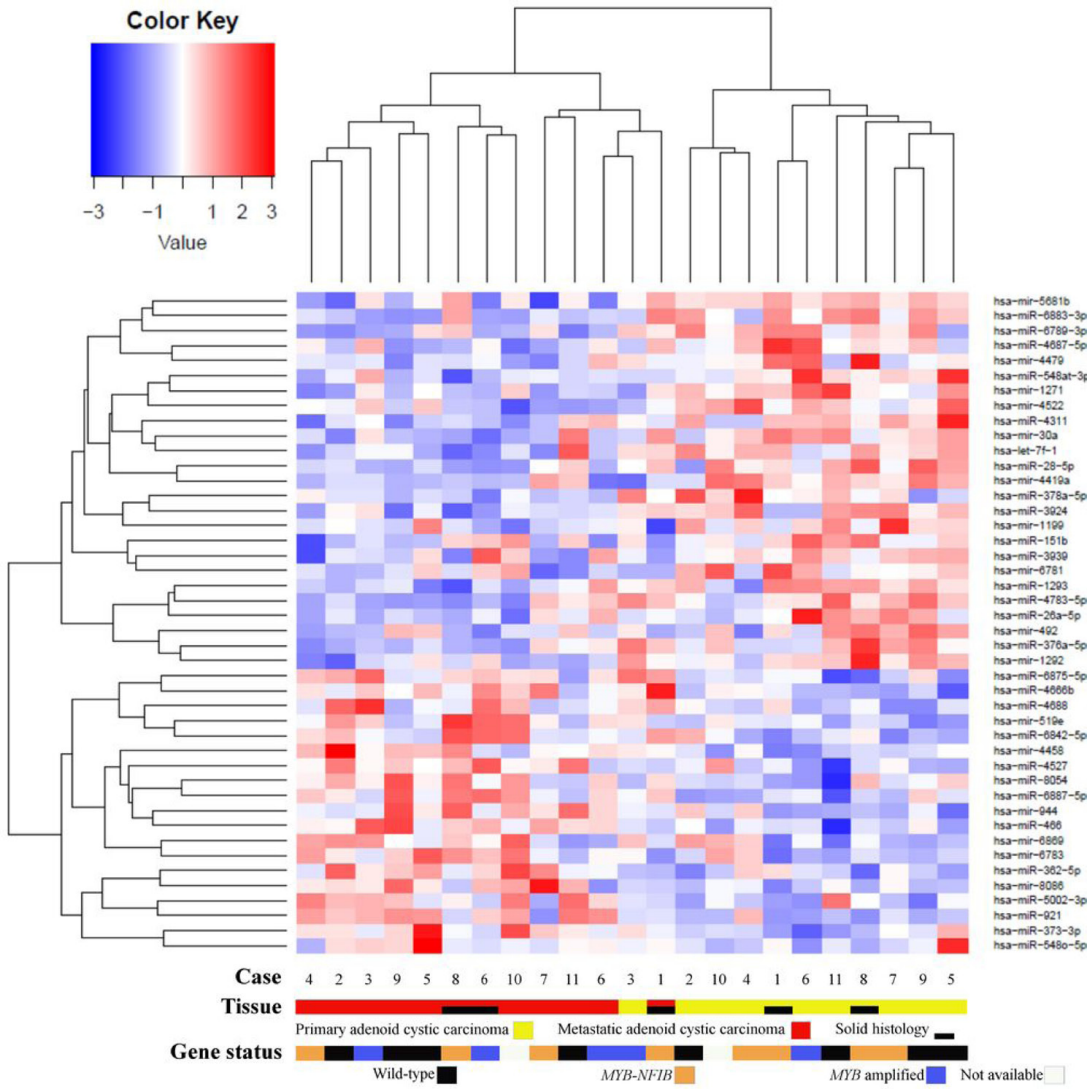
Heatmap of microRNA expression in primary adenoid cystic carcinoma and metastatic adenoid cystic carcinoma The right main cluster consisted of 10/11 primary salivary gland adenoid cystic carcinoma. The left main cluster included 11/11 metastases and the remaining primary tumor. *P*-value < 0.01, FDR > 0.05.

Comparing miRNA expressions of primary ACC and normal salivary gland tissue identified one upregulated (hsa-miR-1271-5p) and 7 downregulated (hsa-miR-1199-3p; hsa-mir-6865; hsa-miR-4717-5p; hsa-mir-610; hsa-miR-6878-5p; hsa-mir-519e; hsa-miR-5572) miRNAs (false discovery rate [FDR] < 0.05). Comparing metastases and normal salivary gland tissue, we identified 4 upregulated (hsa-miR-922; hsa-miR-1271-3p; hsa-mir-6790; hsa-miR-6894-3p) and 7 downregulated (hsa-mir-6865; hsa-miR-1199-3p; hsa-miR-4717-5p; hsa-miR-499a-5p; hsa-miR-4790-5p; hsa-mir-3936; hsa-mir-127) miRNAs (FDR < 0.05) (Figure 4). Among these, hsa-miR-1199-3p, hsa-mir-6865, and hsa-miR-4717-5p were downregulated in primary tumors as well as metastases as compared to normal salivary gland tissue. Functional annotation of the differentially expressed miRNAs between primary ACC and normal salivary gland tissue and between metastases and normal salivary gland tissue identified their involvement in regulating genes in several Kyoto Encyclopedia of Genes and Genomes (KEGG) categories, including metabolism, migration, and signal transduction pathways (Supplementary Tables 3 and 4). When comparing primary ACCs and metastases as groups, there were no significantly differentially expressed miRNAs. Hierarchical clustering including primary and metastatic ACC as well as normal salivary gland tissue placed normal salivary gland together, with the dendrogram including all but one specimen (Figure 5). Primary and metastatic lesions were scattered in the left main cluster and only in one patient (case 7) did the primary tumor and metastasis cluster together (Figure 5). Repeating the analysis including only primary ACC and metastases and re-normalizing data, there was still no difference (FDR > 0.05) but hierarchical clustering by a *p* < 0.01 cut-off for differential expression placed all metastases in one main cluster of the dendrogram and 10/11 primary ACCs in the other main cluster (Figure 6). Resampling did not identify significant differences between the clusters with or without the inclusion of normal salivary gland tissue, but normal salivary gland tissue separated with the highest values (Supplementary Figures 1 and 2).

### MicroRNA expression profiling of primary tumors from patients with and without subsequent recurrence

When comparing the miRNA expression of the primary ACCs along with 11 additional patients who subsequently developed metastases (total *n* = 22) and primary ACCs from patients who remained free of metastases (*n* = 25), no miRNA was differentially expressed at FDR < 0.05.

## DISCUSSION

The clinical course of patients with ACC is unpredictable and once metastatic disease presents, the treatment options are limited as surgical resection is far from always curative and effective targeted treatments are lacking [16, 17]. These features are illustrated in the series presented here as 3/11 patients with subsequent metastatic disease were initially diagnosed with only stage I disease, 5/11 patients died of metastatic disease within the follow-up period, and 3/11 experienced additional distant recurrence following otherwise complete metastasectomies (Table 1).

Over recent decades, genetics has continued to provide insights into the causes of human cancer through the identification of genetic aberrations underlying different malignant phenotypes. But while some cancers are heterogeneous in their genetic background, the identification of recurrent genetic alterations in several types of salivary gland tumors has greatly increased the understanding of these relatively rare entities [18]. In ACC, *NOTCH1* mutations have been shown to be enriched in recurrent and metastatic tumors and mutations in NOTCH pathway genes characterize a small subset of cases with solid histology and a particularly aggressive clinical course [19, 20]. Aside from this, genetics has otherwise not contributed to an understanding of the main cause of ACC mortality, i.e. metastatic spread. Recurrent gene fusions involving *MYB*, *MYBL1*, and *NFIB* are hallmarks of ACC, and we demonstrate the preservation of *MYB* and *NFIB* rearrangements between the primary ACC and the metastases, consistent with the fundamental role of these genes in ACC formation [11]. Interestingly, we found amplification of the 3′ as well as 5′ parts of *MYB* (case 6 and 3, respectively) which were retained in the metastatic cells. While the 5′ part of the *MYB* gene hosts the DNA binding and transactivation domains, the 3′ part of *MYB* hosts the negative regulatory domain that is consistently lost in the *MYB-NFIB* fusion [21]. Therefore, amplification of the 5′ part of *MYB* in case 3 goes well in hand with the oncogenic properties of this part of the *MYB* gene, while the significance of isolated amplification of the 3′ part of *MYB* in case 6 is unclear. Also, we found diffuse expression of MYB protein in all cases, including those without abnormalities of *MYB* as characterized with FISH, which further underline the importance of this protein in ACC biology (Figure 3B). While elucidating the mechanism responsible for MYB overexpression is beyond the scope of this study, others have shown the translocation of various super-enhancer sequences to result in MYB overexpression [8].

Targeted treatment directed by cancer genomics face substantial complexity with the concept of intratumoral heterogeneity, and a study by Liu *et al*. demonstrated this phenomenon in ACC, although to a low extent as compared to other carcinomas [11, 22]. Additional complexity arises when also considering the metastatic compartment of malignant disease, but accounting for this is of paramount importance, since eradication of this cell population is a prerequisite for a sustainable effect of targeted treatment [22]. Liu *et al*. demonstrated that mutations exclusive for metastatic lesions (mainly lymph node metastases) were indeed present in ACC, and to a lesser extent in synchronous as compared to metachronous metastases [11]. In the present study, all metastases were metachronous and most were located at the clinically most relevant anatomical sites for ACC patients, i.e. lung and liver. Using targeted next-generation sequencing, we identified mutations exclusive for the metastatic lesions in 3/11 patients but also mutations exclusive for the primary lesions in another three patients (Table 3). While mutations found exclusively in the metastatic lesions of case 3, 6, and 8 could represent mutations involved in the metastatic phenotype, the significance of the mutations found exclusively in the primary lesions of case 1, 5, and 9 are more likely to be passenger mutations (Table 3). This latter statement is supported by the low frequencies of these mutations (9.3–11.5%). Although the degree of intratumoral heterogeneity in ACC has been shown to be present only to a minor extent, it must be noted that our results rests on the repertoire of mutations identified in 1mm tumor biopsies and not the entire tumor. Therefore, it is possible that the frequencies of different mutations could differ in other areas of the same tumor. In the cases with mutations found exclusively in the metastatic lesions these mutations were all present in high frequencies, but it is not unlikely that the same mutation could be found in the primary lesion, although probably at a lower frequency, if another region had been sampled. Therefore, although we employed a targeted sequencing panel, we confirmed the heterogeneous genomic landscape of paired primary and metastatic ACC. However, in contrast to the tumor-defining oncogenic driver function of ACC gene fusions, interpreting the significance of point mutations in ACC is not an easy task. This is exemplified by a comparison of our findings with datasets from 207 primary salivary gland ACCs and 7 ACC metastases from cBioPortal [23]. In this dataset, mutations in *APC*, *FGFR2*, *HRAS*, *PIK3CA*, and *TP53* were previously reported but were all different from the mutations we found in our material (Supplementary Table 5). However, in accordance with previous studies, mutations in *NOTCH1* tended to cluster in the proline-glutamic acid-serine-threonine (PEST) domain, similar to the activating mutations in T-cell acute lymphoblastic leukaemia (Supplementary Table 5) [11, 19, 20, 24]. Furthermore, demonstrating the diversity of the mutational repertoire involved in the metastatic process is the identification of distinctly different oncogenic hotspot mutations in the lung and liver metastases in case 6. Importantly, the *PIK3CA* in case 6 and *NRAS* in case 1 and 3 are rational candidates for off-label targeted treatments and the *NOTCH1* mutation in case 1 is eligible for inclusion in an ongoing clinical trial for advanced salivary gland cancers (clinicaltrials.gov identifier: NCT020697309) (Table 3). Collectively, this underscores the need for extensive sampling of ACC patients in the context of clinical trials.

While genetics explains the events leading to ACC formation mainly in the form of fusion genes, the low rate of mutations exclusive for the metastatic lesions presented here makes alternative biological mechanisms likely to be involved. As previous *in vitro* studies on the involvement of miRNA in the metastatic progression of ACC has been hampered by cross-contamination, data from human samples has not been available until now [15]. By global miRNA expression profiling we identified miRNAs differentially expressed between primary ACC and metastases as compared to normal salivary gland tissue but no difference between primary and metastatic lesions (Figure 4). Some of these miRNAs were exclusive for primary and metastatic ACCs, but three poorly characterized miRNAs were downregulated in both groups (Figure 4). The significance of these three miRNAs is currently uncertain. Interestingly, functional annotation showed the miRNAs dysregulated in metastases to target genes involved in functions of key importance for the metastatic phenotype, with the most significant function being extracellular molecule interaction (Supplementary Table 4). Normal salivary gland tissue separated from ACC in hierarchical clustering but primary ACCs and metastases were intermingled, with only one case having primary tumor and metastasis placed adjacently (Figure 5 and Supplementary Figure 1). Although this gives an impression of primary tumors and metastases being similar, the inclusion of three different groups with a relatively low number of samples could obscure possible patterns. Although not reaching statistical significance, there was a near complete separation between primary ACC and metastases when normal salivary gland tissue was left out of the clustering analysis (Figure 6). Functional annotation of the miRNAs in Figure 6 identify these to be involved in a large number of metabolic functions, signaling pathways, and as being involved in other types of cancer (Supplementary Table 6). While this finding is intriguing, it must be interpreted with caution as it still relies on non-significant differences and needs further validation. Alternatively, given the highly diverse spectrum of somatic mutations in ACC shown here as well as in other studies, it is possible that ACC is a mainly fusion-driven disease with otherwise diverse molecular changes separating primary and metastatic lesions, including miRNA expression [7, 11, 18, 19, 22].

The acquirement of different somatic mutations on top of the *MYB-NFIB* fusion has been demonstrated to occur during high-grade transformation of breast ACC, which also included mutations in *NOTCH1* and *FGFR1* [25]. Also in breast cancer, remarkably similar miRNA and global gene expression profiles have been found between paired primary breast carcinoma and metastases [26, 27]. In one study these findings extended so far that primary tumors were as similar to their metastases as repeated sampling from the primary tumor, suggesting that the metastatic capability was an inherent feature of the primary tumor and not something unique for the metastatic lesion [27–29]. If this was also the case in salivary gland ACC, this could imply that patients developing metastases had primary tumors that were inherently more “metastatis-like”. Since this would make our comparison of paired primary ACC and metastases futile, we compared the miRNA expression profile of primary ACCs with and without subsequent development of recurrent disease and found no difference in their miRNA expression. This implies that primary ACCs are similar in miRNA expression irrespective of later development of metastases. In direct extension of this consideration it is noteworthy that only the present material of ACC metastases has undergone miRNA expressional profiling, and that comprehensive genetic and miRNA expressional profiling of additional metastatic lesions will extend the understanding of the biological underpinnings of this relatively rare disease.

The complexity in providing a complete molecular landscape of the metastatic process in ACC gives rise to some limitations in our study. Sampling of multiple tumor regions would allow for more solid statements to be made regarding tumor heterogeneity, which would be further strengthened by the use of a more comprehensive sequencing platform for the detection of mutational diversity. Due to the lack of significance between primary and metastatic lesions, the clustering based on miRNA expression may not necessarily reflect the underlying biology, but the rarity of ACCs with available primary and metastatic tissue dictated the sample size which is an inherent limitation to the study of rare diseases.

In conclusion, we present a series of paired primary and metastatic salivary gland ACCs and demonstrate the preservation of *MYB* and *NFIB* aberrations during the course of metastatic progression. Contrasting with the fundamental role of these two genes in ACC, no general pattern of somatic mutations or significant changes in miRNA expression was found in metastatic lesions. Although larger materials could assist in delineating these issues further, our findings support the metastatic process in ACC as being a complex and heterogeneous biological phenomenon.

## MATERIALS AND METHODS

### Patient material

Patients surgically treated for salivary gland ACC and resected metastatic lesion were identified in the Danish Pathology Data Bank (Patobank). Eleven patients with both tissues available were identified, and formalin-fixed and paraffin-embedded (FFPE) specimens were collected from pathology departments all over Denmark. In seven cases, triplet material from primary ACC, metastatic ACC, and normal salivary gland tissue was available. In the remaining four cases only the primary and metastatic tumors were available. For the analysis of miRNA expression in primary tumors only, 11 additional ACCs with subsequent metastases were included along with 25 ACCs that remained free of recurrence. Median follow-up periods for patients with and without subsequent distant failure were 78 months (range: 14–233) and 154 months (range: 72–322), respectively (Supplementary Table 1). None of the patients had received radiotherapy or chemotherapy prior to surgery or biopsy. All cases were reviewed by a head and neck pathologist (TKA) [30–32]. The Regional Ethics Committee (H-6-2014-086) and the Danish Data Protection Agency (REG-94-2014) approved this study.

### Immunohistochemistry

FFPE blocks were sectioned and mounted on coated slides (Dako, Glostrup, Denmark) and stained with H&E according to standard protocols. Tumor sections were deparaffinized using EZ-prep (Ventana Medical Systems^®^, Tucson, AZ, USA) and immunohistochemistry was performed using the Ventana Benchmark Ultra platform (Ventana Medical Systems) as previously described [33]. The following primary antibodies were employed: CD117 (polyclonal, 1:100, Dako (Glostrup, Denmark)), CD56 (clone 1B6, 1:50, Novocastra (Newcastle, UK)), chromogranin A (polyclonal, 1:2000, Dako), CK7 (clone OV-TL 12/30),1:1000, Dako), CK20 (clone KS20.8, 1:400, Dako), ki-67 (clone MIB1, 1:100, Dako), MYB (clone EP769Y 1:150 (AbCam, Cambridge, UK), napsin A (clone IP64, 1:400, Novocastra), and TTF-1(clone SPT24, 1:100, Novocastra). MYB was considered positive when nuclear staining was observed in at least 5% of tumor cells [31]. Positive controls as suggested on datasheets were used. Negative controls omitting the primary antibody were performed for all antibodies.

### Fluorescence *in situ* hybridization

Primary tumor and metastatic lesion were evaluated for rearrangements of *MYB* (Empire Genomics, Buffalo, NY, USA), *MYBL1* (Empire Genomics), and *NFIB* (Empire Genomics) genes with break-apart probes according to the manufacturer's instructions using the HYBrite platform (Abbott Molecular) and counterstained with DAPI II (ZytoVision GmbH, Bremerhaven, Germany) as previously described [31]. One hundred nuclei were counted, and only nuclei where the entire nuclear membrane could be visualized were scored. Break-apart signals in ≥ 10% of cells was considered to represent rearrangement [31]. Amplification was defined as a red/green or green/red signal ratios ≥ 2 signals for 3′ and 5′ amplification, respectively. Amplification was defined as a red/green or green/red signal ratio of ≥ 3 in in more than 10% of tumor cells for 3′ and 5′ amplification, respectively.

### DNA extraction and targeted next-generation sequencing

DNA was extracted from one 1 mm core obtained from the original FFPE block after identification of a representative tumor area from an H&E slide in order to minimize admixture of stromal cells and consequently a high tumor cell content, as previously described [34]. Briefly, targeted NGS was carried out on the Ion PGM™ System (Thermo Fisher Scientific, Walham, MA, USA) with the Ion AmpliSeq Cancer Hotspot Panel version 2 (Thermo Fisher Scientific) according to the manufacturer's instructions. The Ion AmpliSeq Cancer Hotspot Panel v.2 covers approximately 2,800 mutational hotspots in 50 cancer-related oncogenes and tumor-supressor genes. Library preparation was performed from 10 ng of purified DNA using Ion AmpliSeq Library kit 2.0 according to manufacturer's instructions (Thermo Fisher Scientific) and using the Ion PGM Hi-Q view kit (Thermo Fisher Scientific) as template kit for the IonChef (Thermo Fisher Scientific). Sequencing was carried out using Ion PGM Hi-Q view Sequencing kit with the Ion 316™ Chip v2 (Thermo Fisher Scientific). Median number of mapped reads in normal salivary gland, primary tumor, and metastasis were 296,709, 215,455, and 468,226, respectively. Median allele coverage in normal salivary gland, primary tumor, and metastasis were 1,286, 735, and 2,097, respectively. Sequencing statistics are given for each sample in Supplementary Table 7. Variant calling was conducted using the Torrent Variant Caller v4.6 of the Torrent Suite™ Software (Thermo Fisher Scientific) using default settings. Variants were evaluated according to the American College of Medical Genetics and Genomics (ACMG) guidelines using the ClinVar database (https://www.ncbi.nlm.nih.gov/clinvar/docs/acmg/), COSMIC (http://cancer.sanger.ac.uk/cosmic), and by literature search (https://www.ncbi.nlm.nih.gov/pubmed) [35]. Mutant reads were not allowed in the normal tissue. A mutation frequency of 5%, with equal ± strand distribution, was regarded as mutation positive given that DNA was extracted from cores and not whole sections.

### RNA extraction

RNA was extracted from one 1mm core as described above. In case 9, only one pulmonary metastasis had sufficient tissue left. The QiaCube (Qiagen, Valencia, CA, USA) was used for automated isolation of miRNA with the miRNeasy FFPE Kit (Qiagen) according to the manufacturer's instructions [36]. Total RNA concentration was measured using the NanoDrop ND-1000 (Thermo Scientific, Wilmington, DE, USA) and ranged from 366–1536 ng/μl with A260/A280 ratio ranging from 1.8–2.6, indicating high nucleic acid purity.

### MicroRNA array

The Affymetrix miRNA 4.1 array platform (Affymetrix, Santa Clara, CA, USA) was used, covering all entries in Sanger miRbase database v.20, including 2,578 mature and 2,025 immature human miRNAs with a dynamic range of 4 logs. For miRNA analysis, 300 μg of total RNA was labelled with the FlashTag Biotin HSR RNA Labelling kit (Affymetrix) according to the manufacturer's instructions. All cases were run in a single batch to avoid batch variation. Array plates were washed, stained, and scanned on the GeneTitan Instrument (Affymetrix). Raw data are available from the journal website as Supplementary Table 8.

### MicroRNA data analysis

The raw miRNA expression data was normalized using the quantile normalization using the free R package *preprocessCore* [37]. The normalized data was log transformed with base 2 to ensure normal or approximately normal distribution before statistical modelling. Considering the fact that multiple samples (normal salivary gland tissue, primary ACC and metastases) were taken from each patient, we fitted the linear mixed-effect models with miRNA expression levels as the dependent variable, sample grouping (normal, primary, metastasis) as the fixed effect variable, and patient ID as the random effect variable. In fitting the models, normal tissue was defined as the reference group. The mixed-effect models were fitted using the R package *lmerTest*. Differential expression analysis of primary tumors from patients with and without subsequent metastases was done by fitting a regression model that regresses miRNA expression on metastasis status equivalent to a student *t*-test. *P* values from the statistical analysis were adjusted for multiple testing by calculating the FDR using the R function *p.adjust()* defining *method=”fdr”* [38]. The R scripts used for data analysis are available upon request. We applied clustering analysis on the significant miRNAs using the heatmap.2 function in the R package gplots. The default distance algorithm set as “Euclidean” distance was used to compute the distances between clusters. The miRNAs with FDR < 0.05 were defined as statistically significant. Bootstrap resampling was used to generate probability values as implemented in *pvclust* [39].

### Pathway analysis and functional annotation

Pathway analysis of miRNAs was performed with DIANA-miRPath v3.0 software available from http://snf-515788.vm.okeanos.grnet.gr/ [40]. The DIANA-TarBase v7.0 was selected as the miRNAtarget prediction algorithm [41]. For functional annotation, the Kyoto Encyclopedia of Genes and Genomes (KEGG) pathways were used and enrichment score for KEGG pathways presented by –ln (*p* value) [42].

## SUPPLEMENTARY MATERIALS FIGURES AND TABLES







## Abbreviations

ACC: adenoid cystic carcinoma
miRNA: microRNA
FISH: fluorescence *in situ* hybridization
NGS: next-generation sequencing
PEST domain: proline-glutamic acid-serine-threonine domain
FFPE: formalin-fixed and paraffin-embedded
FDR: false discovery rate
KEGG: Kyoto Encyclopedia of Genes and Genomes
