# Supplementary material for: Genetic rearrangements, hotspot mutations, and microRNA expression in the progression of metastatic adenoid cystic carcinoma of the salivary gland
Source: Oncotarget. 2018 Apr 13;9(28):19675–87. doi: 10.18632/oncotarget.24800 (PMC5929417; doi:10.18632/oncotarget.24800)
Supplement: Supplementary file 1 [file oncotarget-09-19675-s001.pdf]

Genetic rearrangements, hotspot mutations, and microRNA expression in the progression of metastatic adenoid cystic carcinoma of the salivary gland

SUPPLEMENTARY MATERIALS

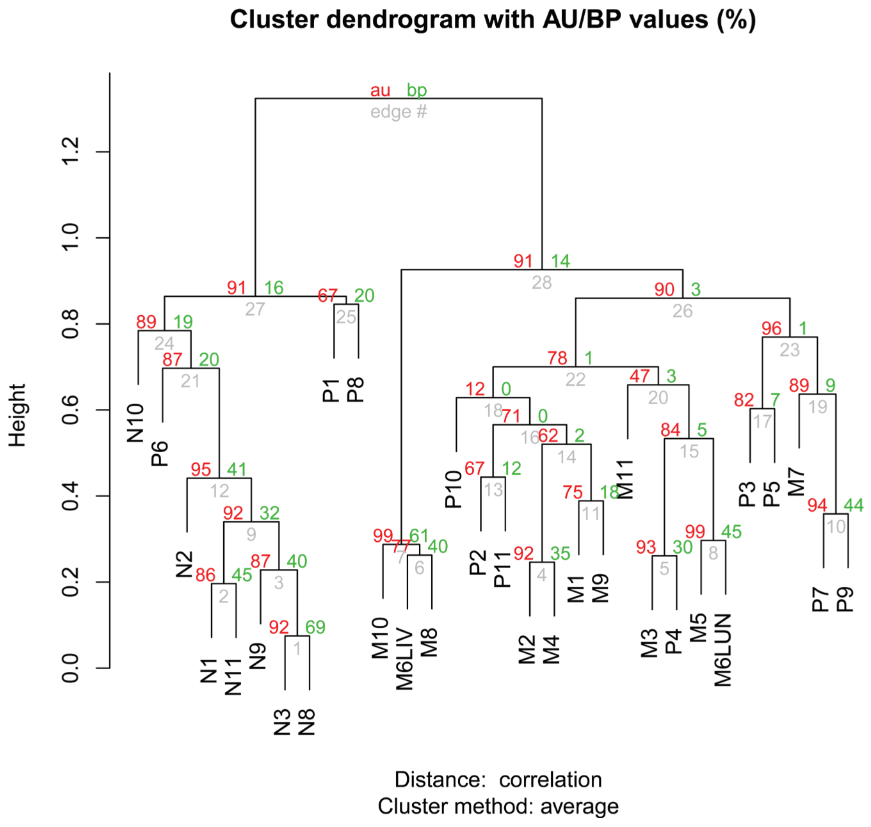

Supplementary Figure 1: Bootstrapping using pvclust including primary tumors, metastases, and normal salivary gland tissue.

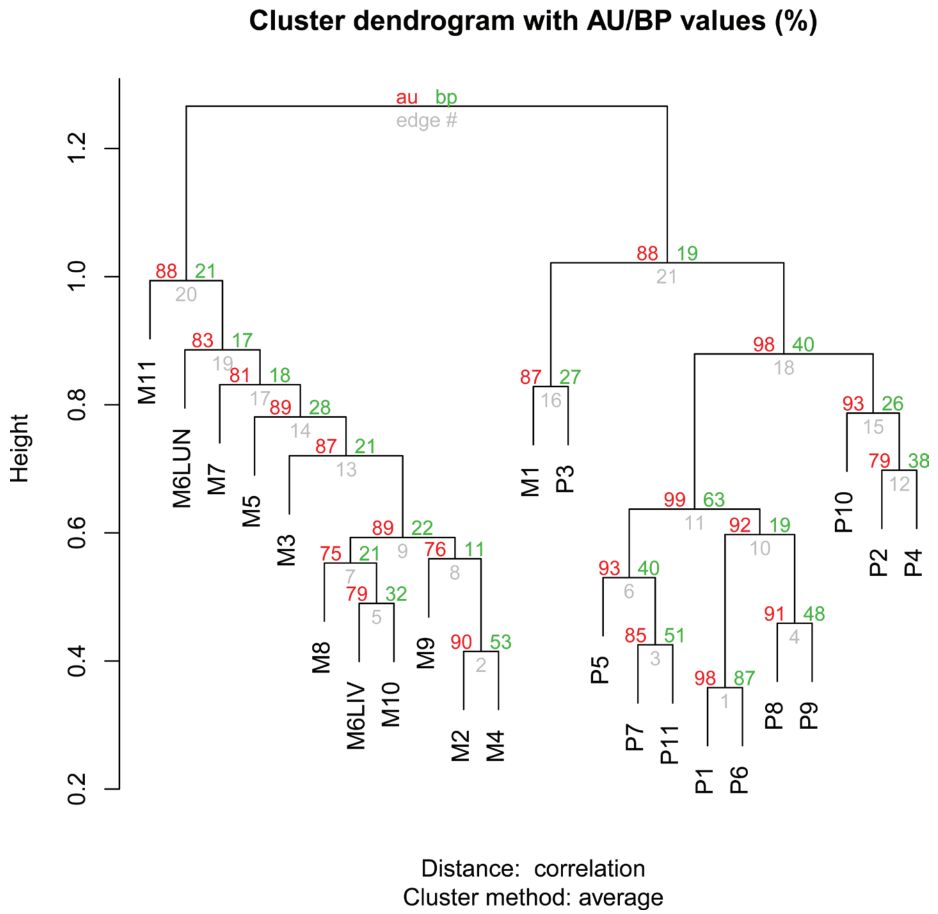

Supplementary Figure 2: Bootstrapping using pvclust including primary tumors and metastases.

Supplementary Table 1: Characteristics of patients without recurrence and all patients with distant metastases

		No recurrence (n = 25)		Distant metastases (n = 22)	
Age at diagnosis (years)	Median, (range)	53 (16–82)		45 (36–76)	
Follow-up (months)	Median, (IQR)	154 (72–322)		78 (14–233)	
		n	%	*n*	%
Site	Parotid gland	7	28	5	22.7
	Submandibular gland	14	56	8	36.4
	Sublingual gland			3	13.6
	Sinonasal tract	1	4	2	9.1
	Palate	2	8	2	9.1
	Base of tongue	1	4	2	9.1
Sex	Female	11	44	13	59.1
	Male	14	56	9	40.9
Status	Alive	22	88	12	54.5
	Dead	3	12	10	45.5
Stage	I/II	20	80	11	50
	III/IV	5	20	11	50
	Missing	0	0	0	0
Margins	Free	9	36	6	27.3
	Close#/Involved	15	60	16	72.7
	Missing	1	4	0	0
Histology	Solid	2	8	4	18.2
	Tubulocribriform	22	88	18	81.8
	Missing	1	4	0	0
Radiotherapy	Yes	24	96	20	90.9
	No	1	4	2	9.1
	Missing	0	0	0	0

IQR= Interquartile range

[*]Discovery cohort: three from the palate, three from buccal mucosa, two from oral tongue; validation cohort: eighteen from the palate, seven from buccal mucosa.

[^]Discovery cohort: one larynx, two base of tongue; validation cohort: three larynx, five base of tongue

[#]Close defined as <5 mm.

Supplementary Table 2: Immunohistochemical staining in paired primary and metastatic adenoid cystic carcinoma

Case	Site	CD56	CD117	Chromogranin A	CK7	CK20	Ki-67 (%)	MYB	Napsin A	TTF-1
1	Primary	−	+	−	+	−	10	+	−	−
	Metastasis	−	+	−	+	−	60	+	−	−
2	Primary	−	+	−	+	−	10	+	−	−
	Metastasis	−	+	−	+	−	10	+	+ (F)	+ (F)
3	Primary	−	+	−	+	−	5	+	−	−
	Metastasis	−	+	−	+	−	5	+	−	−
4	Primary	−	+	−	+	−	15	+	−	−
	Metastasis	−	+	−	+	−	5	+	+ (F)	+ (F)
5	Primary	−	+	−	+	−	10	+	−	−
	Metastasis	−	+	−	+	−	15	+	−	−
6	Primary	−	+	−	+	−	15	+	−	−
	Metastasis (lung)	−	+	−	+	−	20	+	−	−
	Metastasis (liver)	−	+	−	+	−	25	+	−	−
7	Primary	−	+	−	+	−	5	+	−	−
	Metastasis	−	+	−	+	−	10	+	−	−
8	Primary	−	+	−	+	−	35	+	−	−
	Metastasis	−	+	−	+	−	40	+	−	−
9	Primary	−	+	−	+	−	15	+	−	−
	Metastasis	−	+	−	+	−	10	+	−	−
10	Primary	−	+	−	+	−	15	+	−	−
	Metastasis	−	+	−	+	−	5	+	−	
11	Primary	−	+	−	+	−	5	+	−	−
	Metastasis	−	+	−	+	−	10	+	−	

Supplementary Table 3: Functional annotation of microRNAs differentially expressed between normal salivary gland and primary salivary gland adenoid cystic carcinoma according to the Kyoto Encyclopedia of Genes and Genomes (KEGG)

KEGG pathway name (KEGG ID)	KEGG orthology	Number of genes regulated by differentially expressed miRNAs	*p* value
Fatty acid biosynthesis (hsa00061)	Lipid metabolism	1	8.30e-34
Fatty acid metabolism (hsa01212)	Metabolism	3	4.73e-25
Biosynthesis of unsaturated fatty acids (hsa01040)	Lipid metabolism	1	0.003
Glycosaminoglycan degradation (hsa00531)	Glycan biosynthesis and metabolism	2	0.018
Fatty acid elongation (hsa00062)	Lipid metabolism	2	0.020
Metabolic pathways (hsa01100)	Glycan biosynthesis and metabolism	28	0.035
Prostate cancer (hsa05215)	Cancers	8	0.036

Supplementary Table 4: Functional annotation of microRNAs differentially expressed between normal salivary gland and metastatic salivary gland adenoid cystic carcinoma according to the Kyoto Encyclopedia of Genes and Genomes (KEGG)

KEGG pathway name (KEGG ID)	KEGG orthology	Number of genes regulated by differentially expressed miRNAs	*p* value
ECM-receptor interaction (hsa04512)	Signaling molecules and interaction	7	4.14e–5
Other types of O-glycan biosynthesis (hsa00514)	Glycan biosynthesis and metabolism	3	<0.001
Tyrosine metabolism (hsa00350)	Amino acid metabolism	2	0.003
Hepatitis B (hsa05161)	Infectious diseases	13	0.003
Pathways in cancer (hsa05200)	Human diseases	31	0.003
Viral carcinogenesis (hsa05203)	Human diseases	19	0.003
Amino sugar and nucleotide sugar metabolism (hsa00520)	Carbohydrate metabolism	4	0.003
Small cell lung cancer (hsa05222)	Cancers	12	0.005
Huntington’s disease (hsa05016)	Neurodegenerative diseases	10	0.008
Chronic myeloid leukemia (hsa05220)	Human diseases	10	0.008
PI3K-Akt signaling pathway (hsa04151)	Signal transduction	30	0.008
p53 signaling pathway (hsa04115)	Cell growth and death	9	0.011
Thyroid hormone signaling pathway (hsa04919)	Endocrine system	11	0.016
Measles (hsa05162)	Infectious diseases	15	0.016
Cell cycle (hsa04110)	Cell growth and death	14	0.03

Supplementary Table 5: Lollipop plots illustrating the frequency and distribution of *APC*, *BRAF*, *FGFR2*, *HRAS*, *NOTCH1*, *NRAS*, *PDGFRA*, *PIK3CA*, and *TP53* mutations in 207 primary ACCs and 7 metastases available from CBioPortal [1–4]

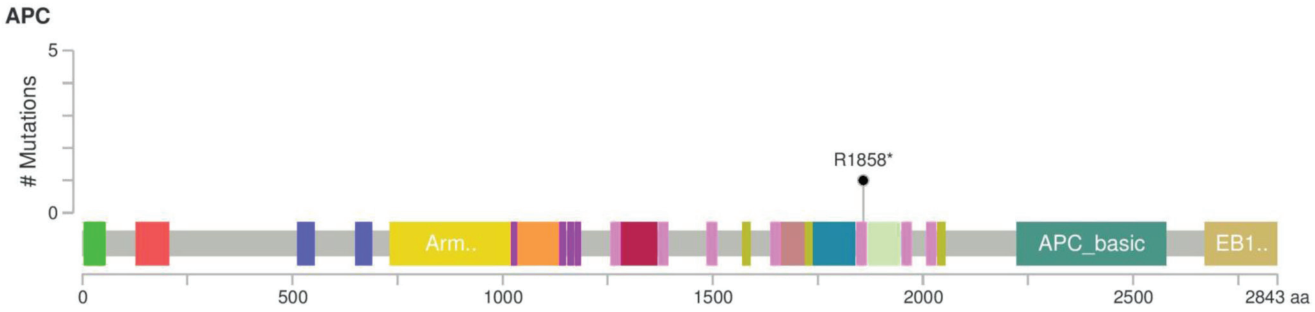

One mutation in one primary tumour.

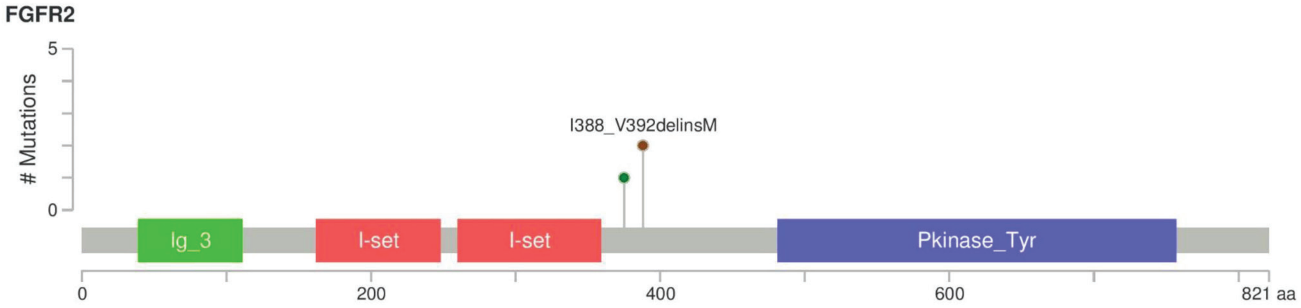

Three mutations in three primary tumours.

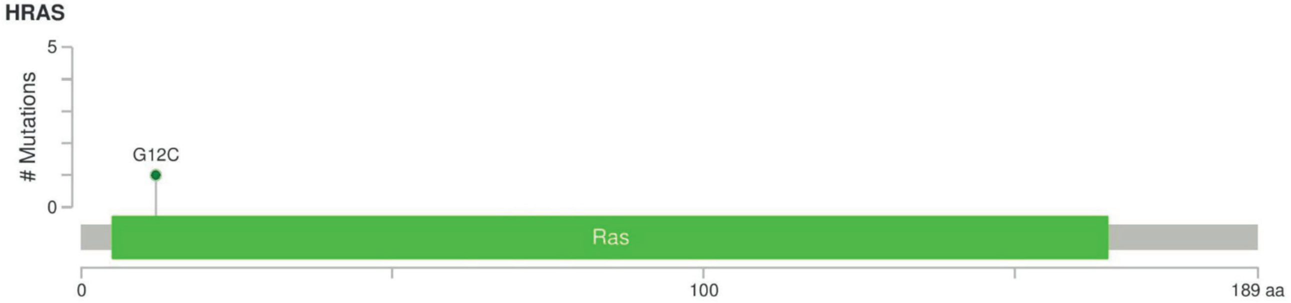

One mutation in one primary tumour.

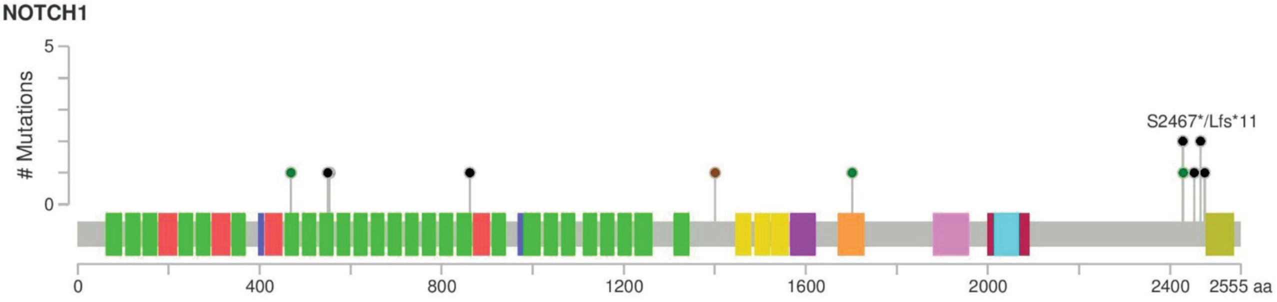

Thirteen mutations in 12 primary tumours. Note the clustering of mutations in the proline-glutamic acid-serine-threonine (PEST) domain to the far right.

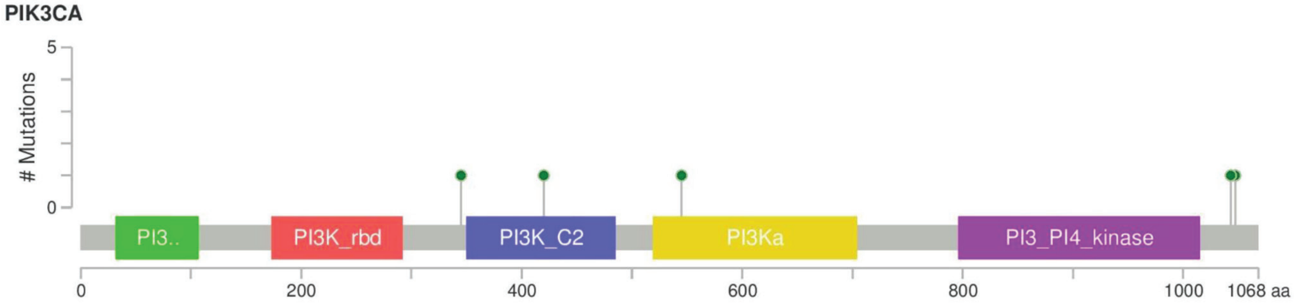

Five mutations in five primary tumours.

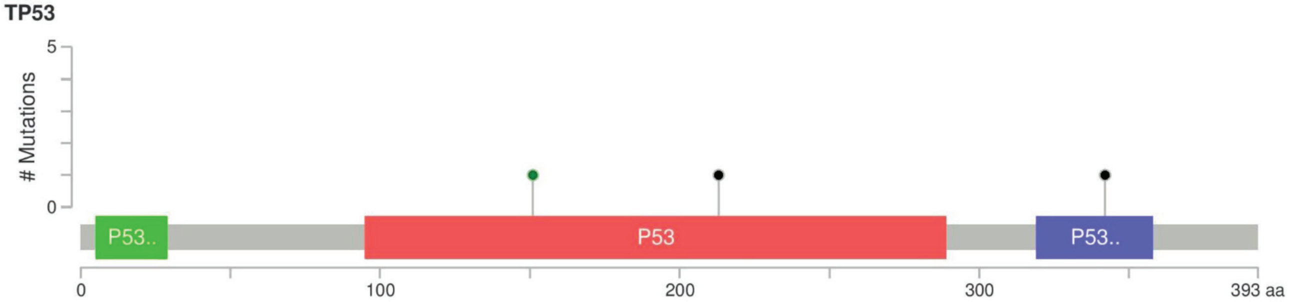

Three mutations in three primary tumours.

Color codes:
Green: missense mutations; black: truncating mutation; brown: inframe mutation.

Supplementary Table 6: Functional annotation of miRNAs in figure 6 according to the Kyoto Encyclopedia of Genes and Genomes (KEGG)

KEGG pathway name (KEGG ID)	KEGG orthology	Number of genes regulated by differentially expressed miRNAs	*p* value
Protein processing in endoplasmic reticulum (hsa04141)	Genetic information processing	76	6.72e–07
Fatty acid biosynthesis (hsa00061)	Metabolism	4	4.20e–07
Viral carcinogenesis (hsa05203)	Cancers	78	1.19e–06
Proteoglycans in cancer (hsa05205)	Cancers	80	1.74e–06
Chronic myeloid leukemia (hsa05220)	Cancers	40	1.94e–06
Oocyte meiosis (hsa04114)	Cell growth and death	28	1.96e–06
Thyroid hormone signaling pathway (hsa04919)	Cancers	52	1.96e–06
Lysine degradation (hsa00310)	Metabolism	23	6.16e–06
FoxO signaling pathway (hsa04068)	Signal transduction	63	1.25e–05
Cell cycle (hsa04110)	Cell growth and death	60	1.37e–05
Colorectal cancer (hsa05210)	Cancers	33	2.18e–05
Hippo signaling pathway (hsa04390)	Signal transduction	56	2.18e–05
Pathways in cancer (hsa05200)	Cancers	146	2.18e–05
RNA transport (hsa03013)	Genetic information processing	73	2.76e–05
Ubiquitin mediated proteolysis (hsa04120)	Genetic information processing	66	2.76e–05
TGF-beta signaling pathway (hsa04350)	Signal transduction	36	3.62e–05
Glioma (hsa05214)	Cancers	31	4.66e–05
Small cell lung cancer (hsa05222)	Cancers	43	7.70e–05
Pancreatic cancer (hsa05212)	Cancers	33	<0.001
mRNA surveillance pathway (hsa03015)		45	<0.001
Prostate cancer (hsa05215)		41	<0.001
p53 signaling pathway (hsa04115)		34	<0.001
Signaling pathways regulating pluripotency of stem cells (hsa04550)		34	<0.001
Prolactin signaling pathway (hsa04917)		33	0.001
Renal cell carcinoma (hsa05211)		32	0.002

Top 25 pathways are shown.

Supplementary Table 7: Next-generation sequencing specifications for each sample. See Supplementary_Table_7

Supplementary Table 8: MicroRNA array data. See Supplementary_Table_8

REFERENCES

1. Ross JS, Wang K, Rand JV, Sheehan CE, Jennings TA, Al-Rohil RN, Otto GA, Curran JC, Palmer G, Downing SR, Yelensky R, Lipson D, Balasubramanian S, et al. Comprehensive genomic profiling of relapsed and metastatic adenoid cystic carcinomas by next-generation sequencing reveals potential new routes to targeted therapies. Am J Surg Pathol. 2014; 38:235–238. https://doi.org/10.1097/PAS.0000000000000102.
2. Mitani Y, Liu B, Rao PH, Borra VJ, Zafereo M, Weber RS, Kies M, Lozano G, Futreal PA, Caulin CA, El-Naggar AK. Novel MYBL1 Gene Rearrangements with Recurrent MYBL1-NFIB Fusions in Salivary Adenoid Cystic Carcinomas Lacking t(6;9) Translocations. Clin Cancer Res. 2016; 22:725–733. https://doi.org/10.1158/1078-0432.CCR-15-2867-T.
3. Ho AS, Kannan K, Roy DM, Morris LGT, Ganly I, Katabi N, Ramaswami D, Walsh LA, Eng S, Huse JT, Zhang J, Dolgalev I, Huberman K, et al. The mutational landscape of adenoid cystic carcinoma. Nat Genet. 2013; 45:791–798. https://doi.org/10.1038/ng.2643.
4. Stephens PJ, Davies HR, Mitani Y, Van Loo P, Shlien A, Tarpey PS, Papaemmanuil E, Cheverton A, Bignell GR, Butler AP, Gamble J, Gamble S, Hardy C, et al. Whole exome sequencing of adenoid cystic carcinoma. J Clin Invest. 2013; 123:2965–2968. https://doi.org/10.1172/JCI67201.
